# Supplementary material for: Multi-functional photonic crystals of modular nanosheets
Source: Nat Commun. 2026 May 26;17:4517. doi: 10.1038/s41467-026-70456-6 (PMC13212688; doi:10.1038/s41467-026-70456-6)
Supplement: Supplementary file 1 — Supplementary Information [file 41467_2026_70456_MOESM1_ESM.pdf]

Supplementary Information

Multi-functional photonic crystals of modular nanosheets

Seiya Yui[1], Takumi Mihara[1], Tomoki Nishimura[1], Yasuo Ebina[2], Takayoshi Sasaki[2] and Koki Sano[1]*

[1] Department of Chemistry and Materials, Faculty of Textile Science and Technology, Shinshu University, 3-15-1 Tokida, Ueda, Nagano 386-8567, Japan.

[2] Research Center for Materials Nanoarchitectonics (MANA), National Institute for Materials Science (NIMS), 1-1 Namiki, Tsukuba, Ibaraki 305-0044, Japan.

Correspondence and requests for materials should be addressed to Koki Sano (koki_sano@shinshu-u.ac.jp).

Supplementary Figures

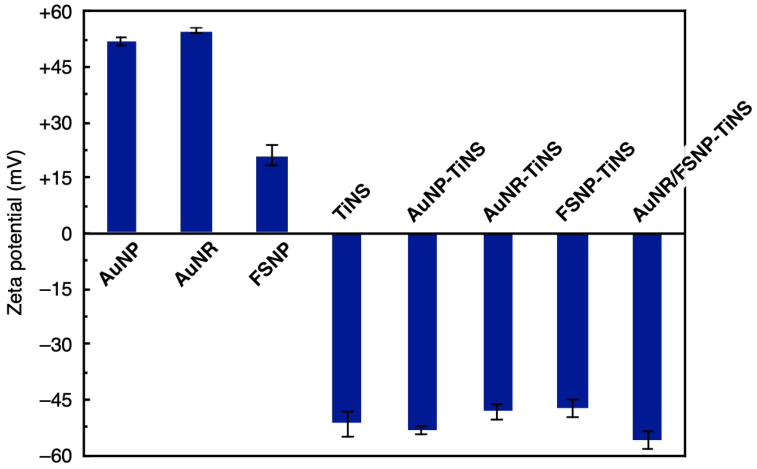

Supplementary Fig. 1 | Zeta potentials of functional nanoparticles and nanosheets.

Zeta potential values of positively charged functional nanoparticles (AuNPs, AuNRs, and FSNPs) and negatively charged nanosheets (TiNSs, AuNP-TiNSs, AuNR-TiNSs, FSNP-TiNSs, and AuNR/FSNP-TiNSs). Data are presented as mean values ± SD (n = 3; the same sample was measured repeatedly).

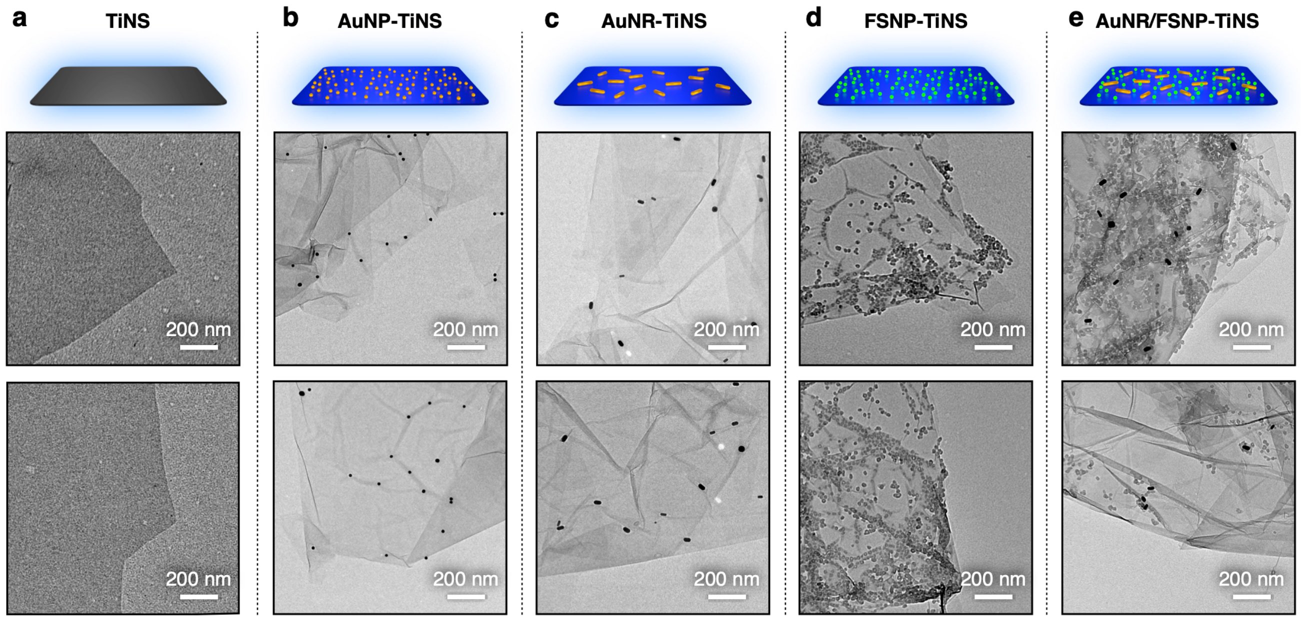

Supplementary Fig. 2 | TEM images of nanosheets.

a–**e**, Transmission electron microscopy (TEM) images of nanosheets (**a**: TiNSs; **b**: AuNP-TiNSs; **c**: AuNR-TiNSs; **d**: FSNP-TiNSs; **e**: AuNR/FSNP-TiNSs).

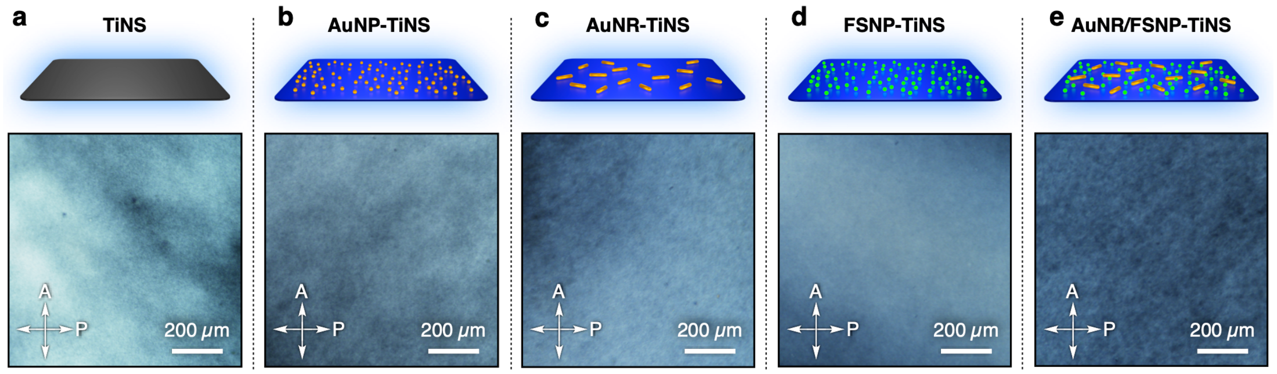

Supplementary Fig. 3 | POM images of aqueous dispersions of nanosheets.

a–**e**, Polarized optical microscopy (POM) images under crossed Nicols of aqueous dispersions of nanosheets at a concentration of 0.20 wt% (**a**: TiNSs; **b**: AuNP-TiNSs; **c**: AuNR-TiNSs; **d**: FSNP-TiNSs; **e**: AuNR/FSNP-TiNSs) in 1-mm-thick quartz cuvettes (40 × 10 × 1 mm).

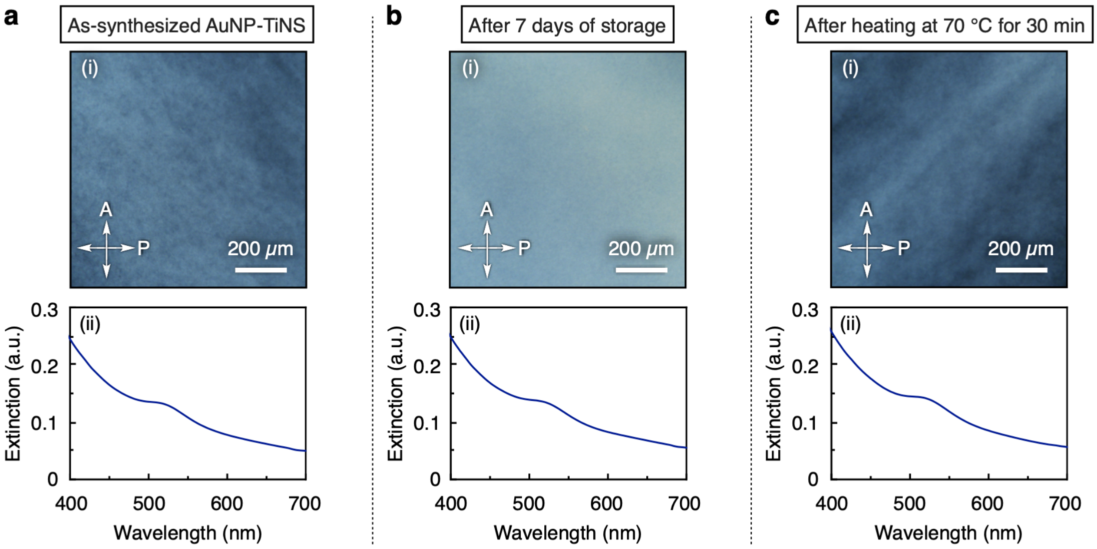

Supplementary Fig. 4 | Long-term and thermal stability of AuNP-TiNS.

a–**c**, (i) Polarized optical microscopy (POM) images under crossed Nicols and (ii) extinction spectra of aqueous dispersions (0.20 wt%) of (**a**) as-synthesized AuNP-TiNSs, (**b**) AuNP-TiNSs after 7 days of storage, and (**c**) AuNP-TiNSs after heating at 70 °C for 30 min in 1-mm-thick quartz cuvettes (40 × 10 × 1 mm).

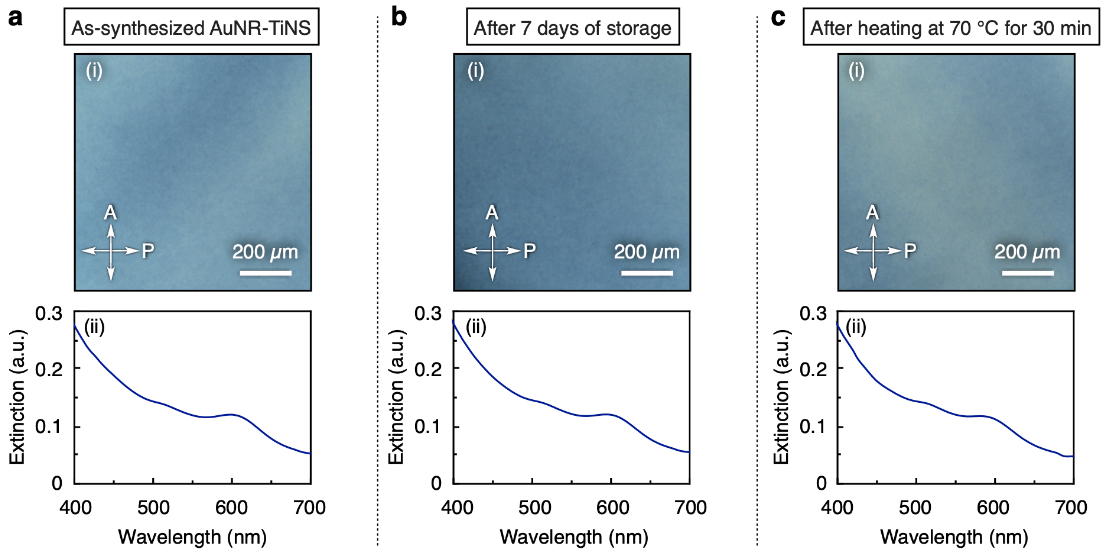

Supplementary Fig. 5 | Long-term and thermal stability of AuNR-TiNS.

a–**c**, (i) Polarized optical microscopy (POM) images under crossed Nicols and (ii) extinction spectra of aqueous dispersions (0.20 wt%) of (**a**) as-synthesized AuNR-TiNSs, (**b**) AuNR-TiNSs after 7 days of storage, and (**c**) AuNR-TiNSs after heating at 70 °C for 30 min in 1-mm-thick quartz cuvettes (40 × 10 × 1 mm).

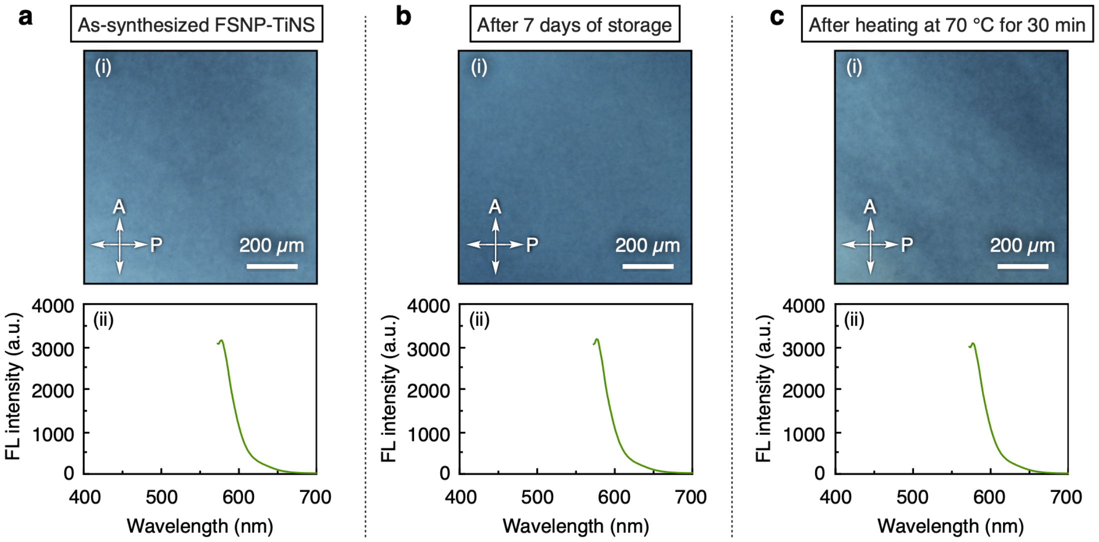

Supplementary Fig. 6 | Long-term and thermal stability of FSNP-TiNS.

a–**c**, (i) Polarized optical microscopy (POM) images under crossed Nicols and (ii) fluorescence spectra of aqueous dispersions (0.20 wt%) of (**a**) as-synthesized FSNP-TiNSs, (**b**) FSNP-TiNSs after 7 days of storage, and (**c**) FSNP-TiNSs after heating at 70 °C for 30 min in 1-mm-thick quartz cuvettes (40 × 10 × 1 mm).

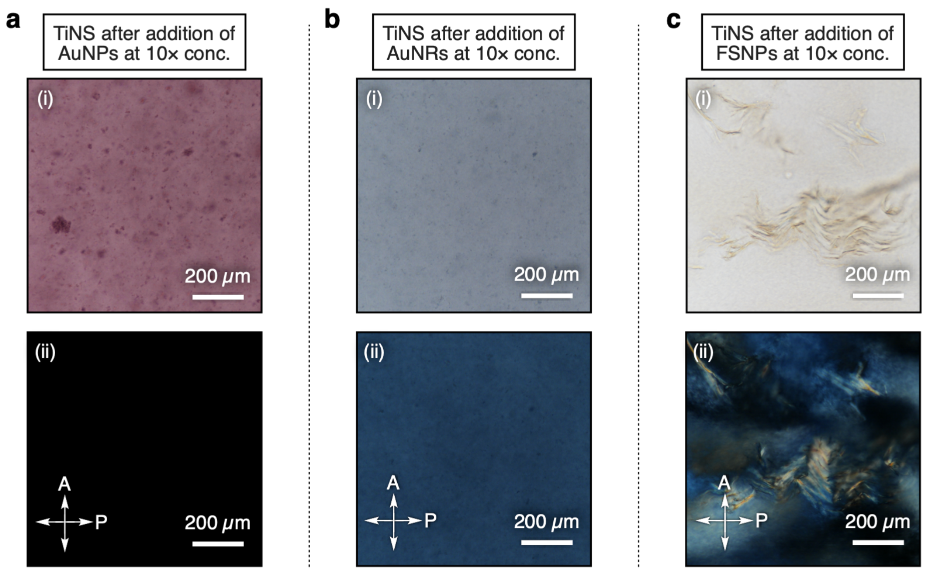

Supplementary Fig. 7 | TiNS dispersions after addition of excessive nanoparticles.
a–**c**, (i) Optical microscopy images and (ii) polarized optical microscopy (POM) images under crossed Nicols of aqueous dispersions of TiNSs (0.20 wt%) in 1-mm-thick quartz cuvettes (40 × 10 × 1 mm) after adding nanoparticles at 10× their respective optimized amounts (**a**: AuNPs; **b**: AuNRs; **c**: FSNPs).

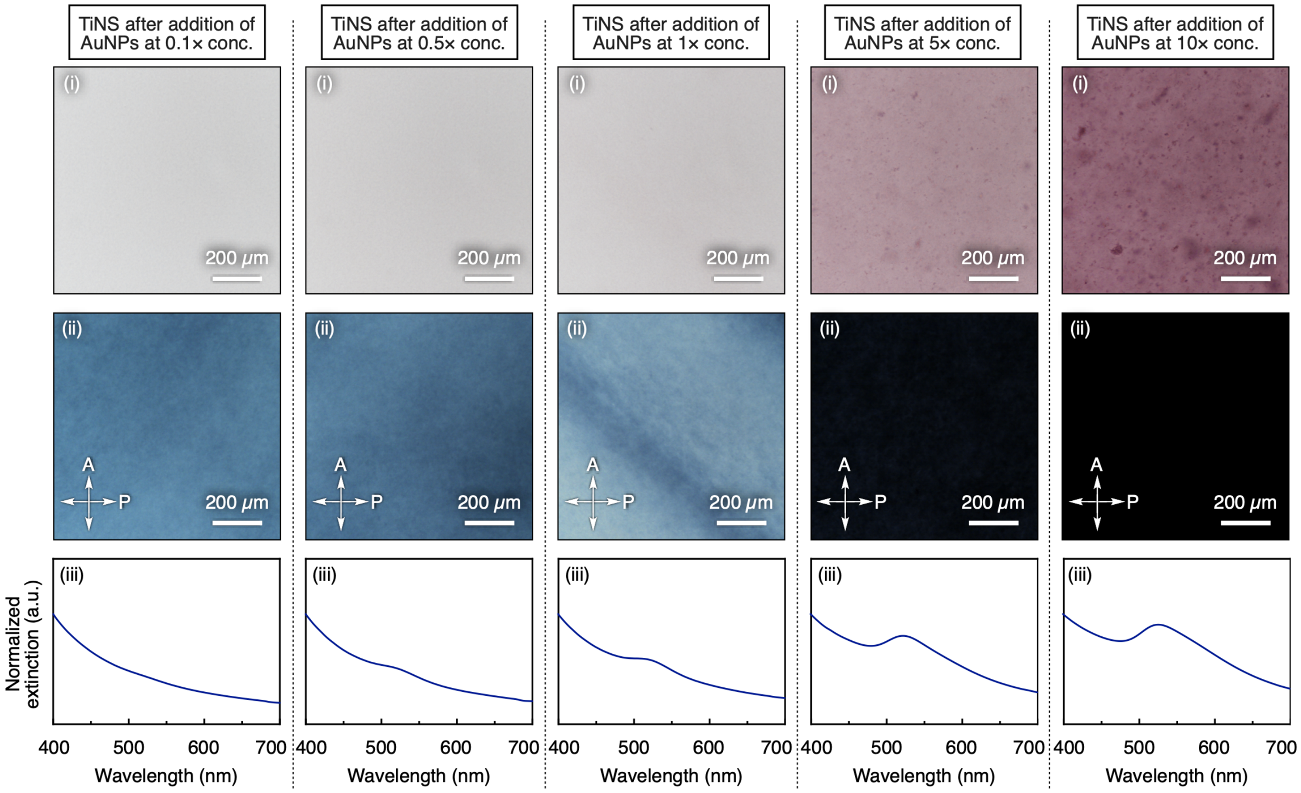

Supplementary Fig. 8 | TiNS dispersions after addition of varying amounts of AuNPs.

(i) Optical microscopy images, (ii) polarized optical microscopy (POM) images under crossed Nicols, and (iii) normalized extinction spectra of aqueous dispersions of TiNSs (0.20 wt%) in 1-mm-thick quartz cuvettes (40 × 10 × 1 mm) after adding AuNPs at 0.1×, 0.5×, 1×, 5×, or 10× the optimized amount.

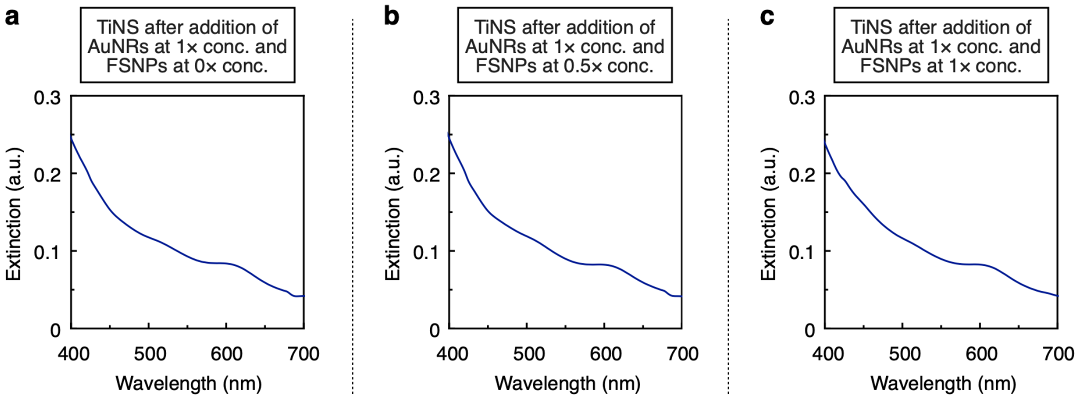

Supplementary Fig. 9 | TiNS dispersions after addition of AuNRs and FSNPs at varying ratios. **a**–**c**, Extinction spectra of aqueous dispersions of TiNSs (0.20 wt%) in 1-mm-thick quartz cuvettes (40 × 10 × 1 mm) after adding AuNRs and FSNPs at different multiples of their respective optimized amounts (**a**: AuNRs 1× and FSNPs 0×; **b**: AuNRs 1× and FSNPs 0.5×; **c**: AuNRs 1× and FSNPs 1×).

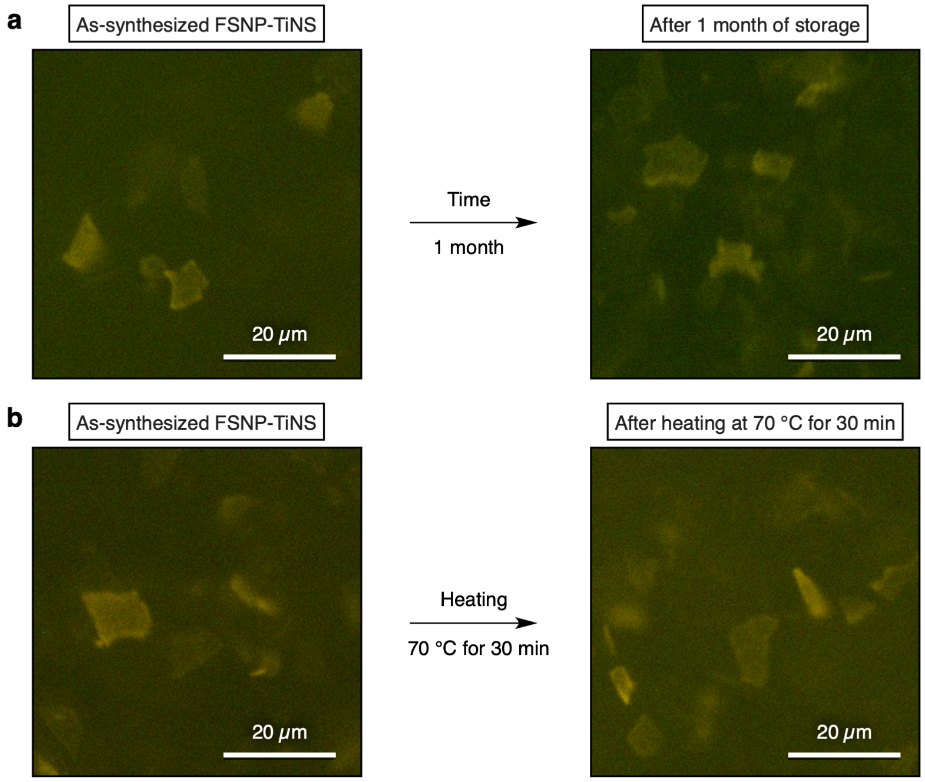

Supplementary Fig. 10 | Fluorescence microscopy images of fluorescent nanosheets.
a,**b**, Fluorescence microscopy images of aqueous dispersions of FSNP-TiNSs (0.0050 wt%) (**a**) before and after one month of storage and (**b**) before and after heating at 70 °C for 30 min.

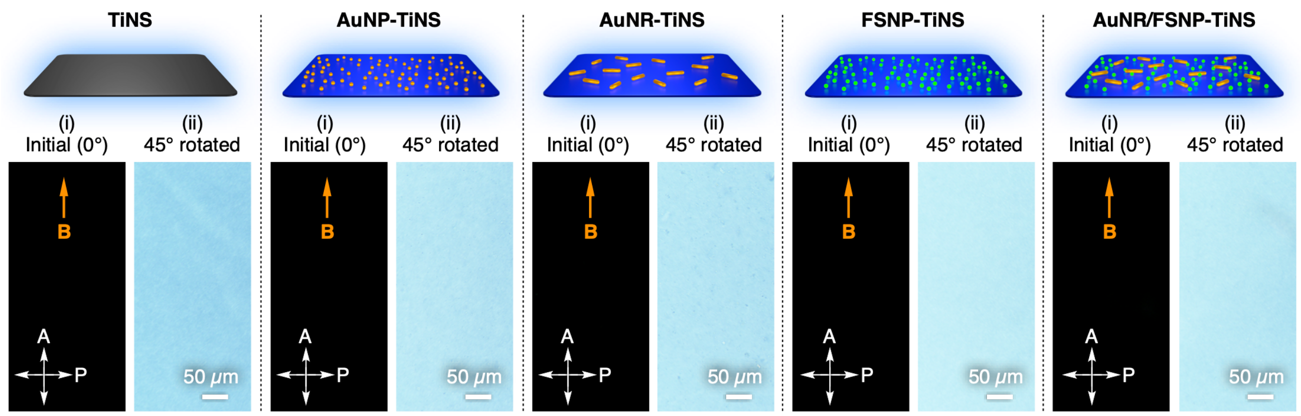

Supplementary Fig. 11 | POM images of magnetically treated nanosheets.

POM images under crossed Nicols of magnetically treated nanosheets at a concentration of 0.050 wt% (TiNSs, AuNP-TiNSs, AuNR-TiNSs, FSNP-TiNSs, and AuNR/FSNP-TiNSs) fixed in hydrogels (i) before and (ii) after sample rotation by 45°.

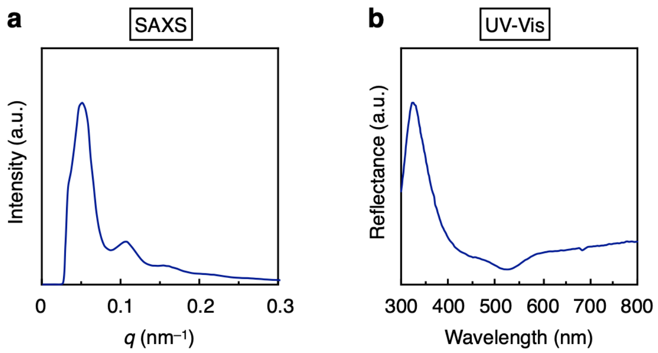

Supplementary Fig. 12 | SAXS and reflection of the photonic crystal of AuNP-TiNSs.
a,**b**, (**a**) 1D small-angle X-ray scattering (SAXS) profile and (**b**) reflection spectrum of the photonic crystal of AuNP-TiNSs (2.0 wt%).

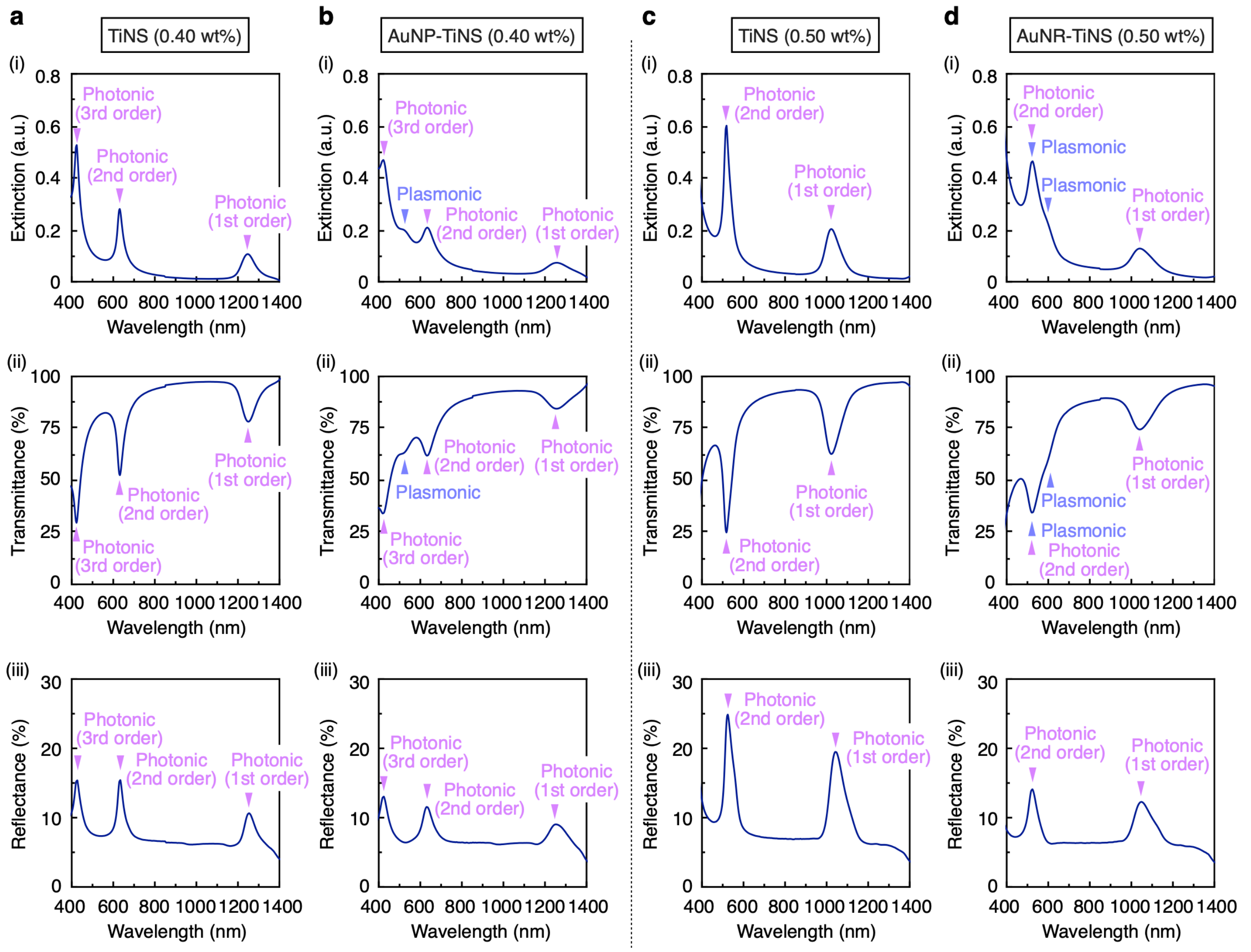

Supplementary Fig. 13 | Optical properties of the photonic crystal of AuNP-TiNSs.

a–**d**, (i) Extinction and (ii) transmittance spectra measured in transmission mode and (iii) reflectance spectra measured in reflection mode of the photonic crystal of pristine TiNSs (**a**: 0.40 wt%; **c**: 0.50 wt%), (**b**) the photonic crystal of AuNP-TiNSs (0.40 wt%), and (**d**) the photonic crystal of AuNR-TiNSs (0.50 wt%) in 1-mm-thick quartz cuvettes (40 × 10 × 1 mm).

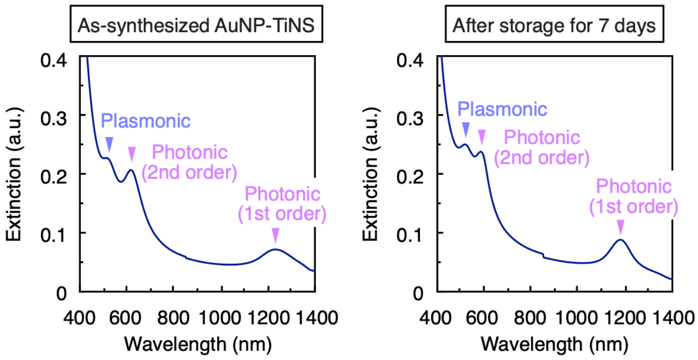

Supplementary Fig. 14 | Long-term stability of the photonic crystal of AuNP-TiNSs.
Extinction spectra of the photonic crystal of AuNP-TiNSs (0.40 wt%) in 1-mm-thick quartz cuvettes (40 × 10 × 1 mm) before and after 7 days of storage.

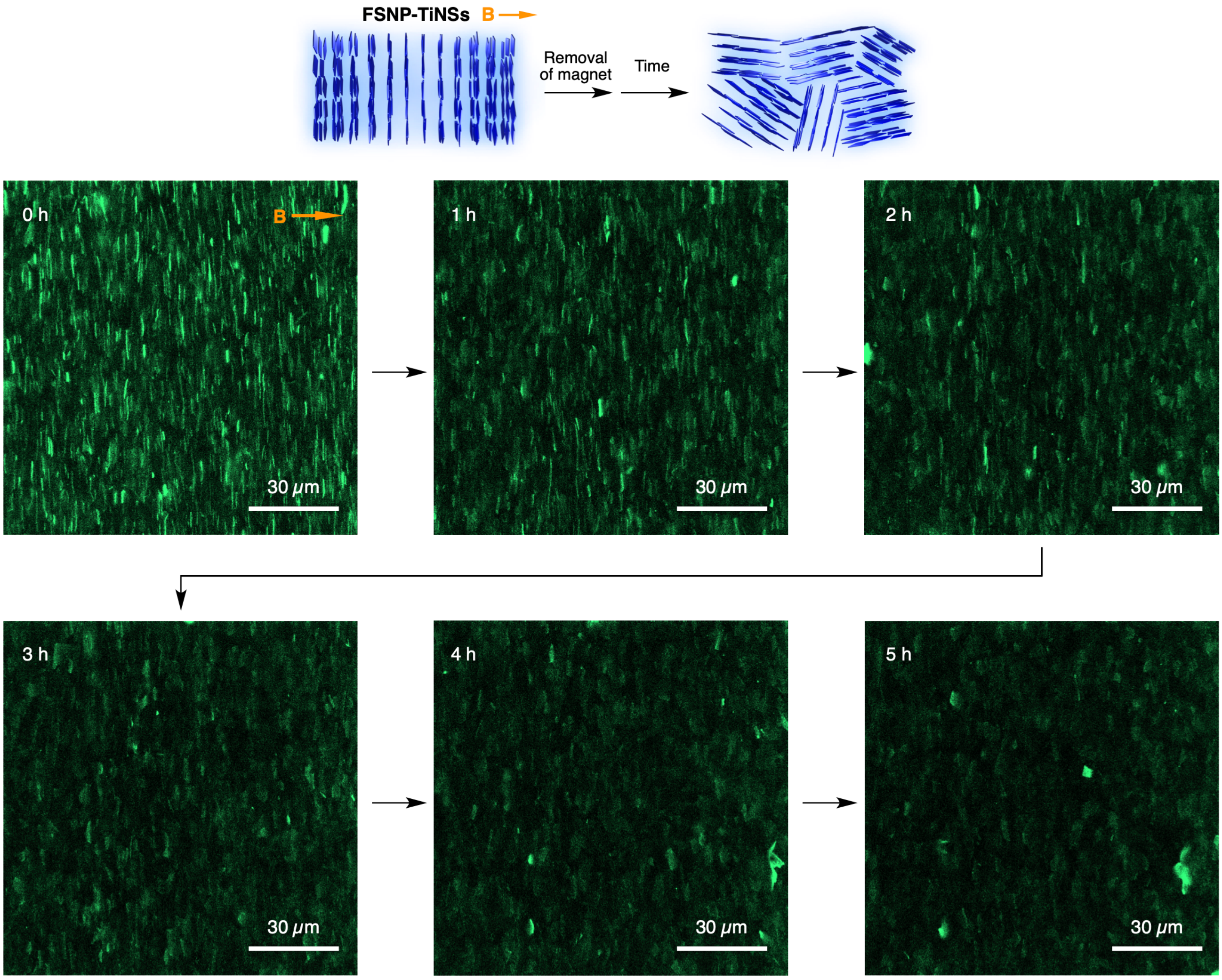

Supplementary Fig. 15 | Time-dependent CLSM image of the magnetically treated photonic crystal of FSNP-TiNSs.

Time-dependent confocal laser scanning microscopy (CLSM) images of the magnetically treated photonic crystal of FSNP-TiNSs (0.40 wt%) in a dispersion state using a 550-nm laser.

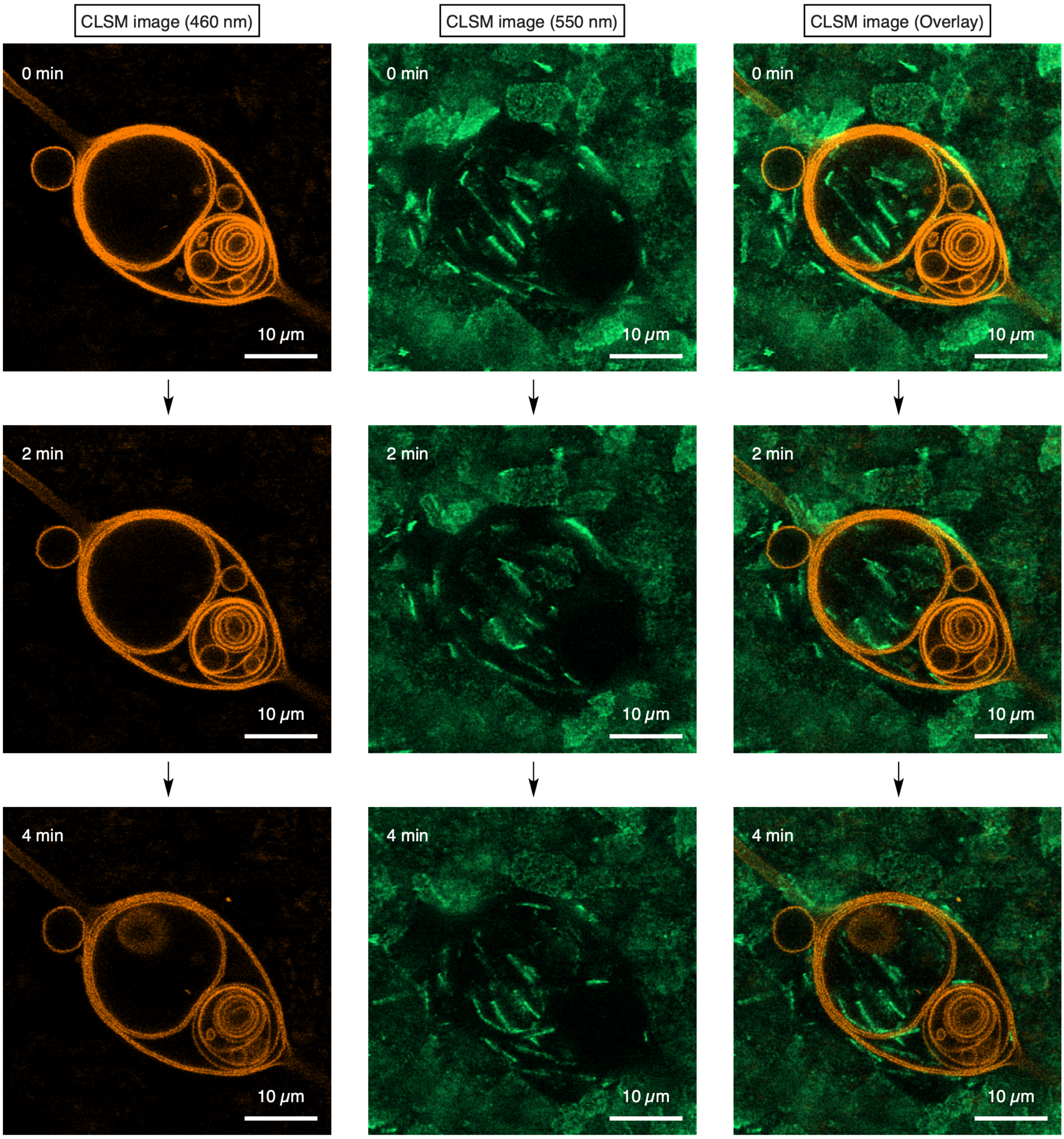

Supplementary Fig. 16 | Time-dependent CLSM image of the photonic crystal of FSNP-TiNSs within giant vesicles.

Time-dependent confocal laser scanning microscopy (CLSM) images of the photonic crystal of FSNP-TiNSs (0.40 wt%) within giant vesicles using a 460-nm laser for the vesicles and a 550-nm laser for the nanosheets.

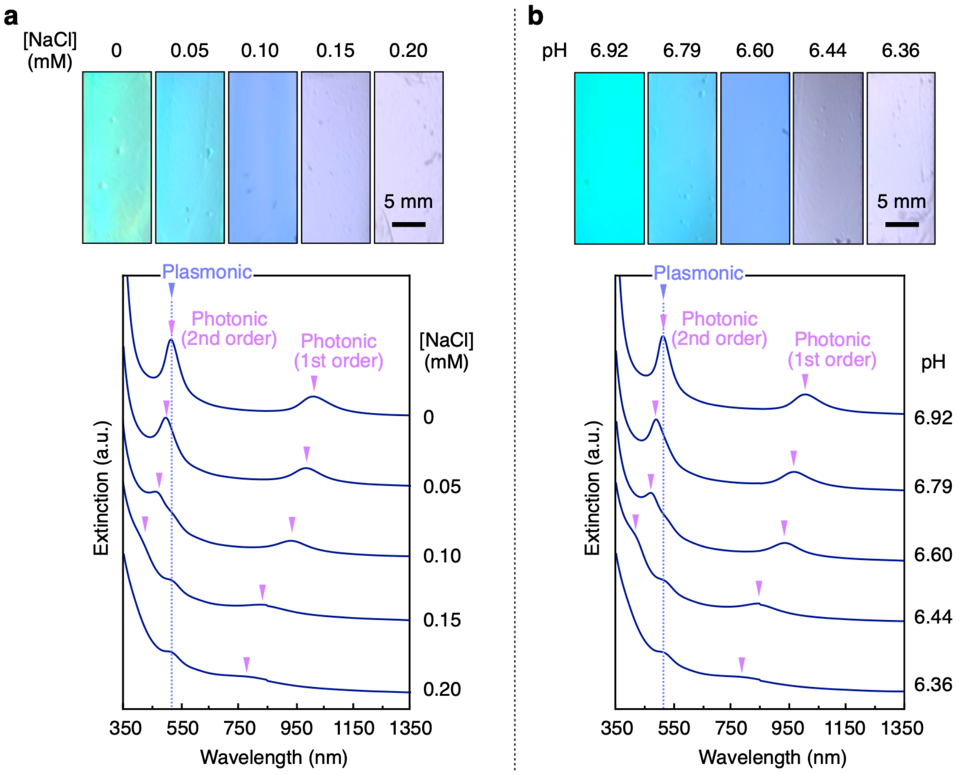

Supplementary Fig. 17 | Structural colors of the photonic crystal of AuNP-TiNSs as a function of ionic concentration and pH.

a,**b**, Optical images (upper) and extinction spectra (lower) of the magnetically treated photonic crystals of AuNP-TiNSs (0.50 wt%) in 1-mm-thick quartz cuvettes (40 × 10 × 1 mm) at (**a**) ionic concentrations ([NaCl] = 0, 0.05, 0.10, 0.15, and 0.20 mM) and (**b**) pH (6.92, 6.79, 6.60, 6.44, and 6.36). The pH was adjusted by adding an aqueous HCl solution.

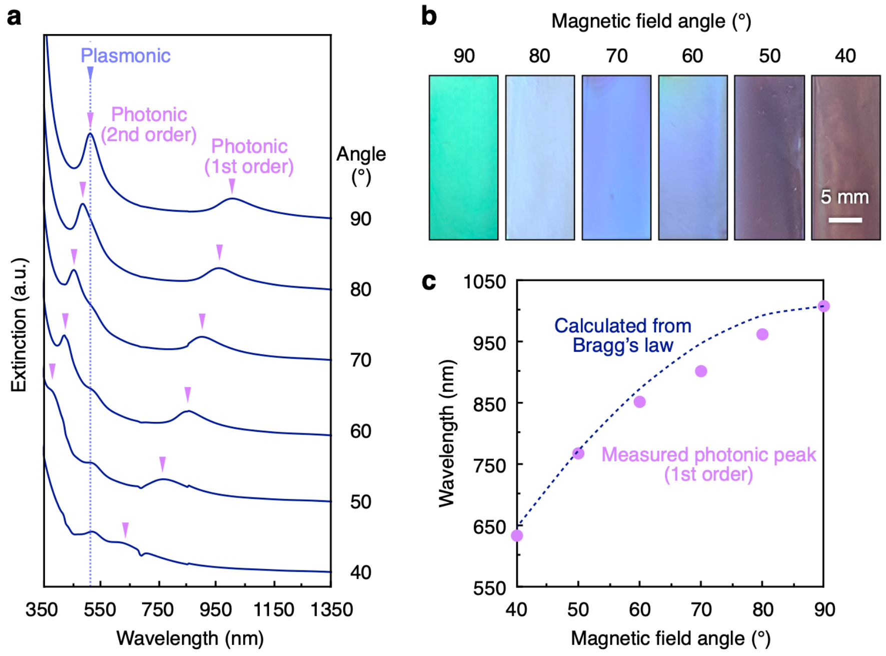

Supplementary Fig. 18 | Structural colors of the photonic crystal of AuNP-TiNSs as a function of magnetic field angle.

a,**b**, (**a**) extinction spectra and (**b**) optical images of the magnetically treated photonic crystals of AuNP-TiNSs (0.50 wt%) in 1-mm-thick quartz cuvettes (40 × 10 × 1 mm) by varying the angle of the applied magnetic field with respect to the cuvette surface. **c**, Observed wavelength of the first-order structural colors and calculated values from Bragg's law as a function of the magnetic field angle.

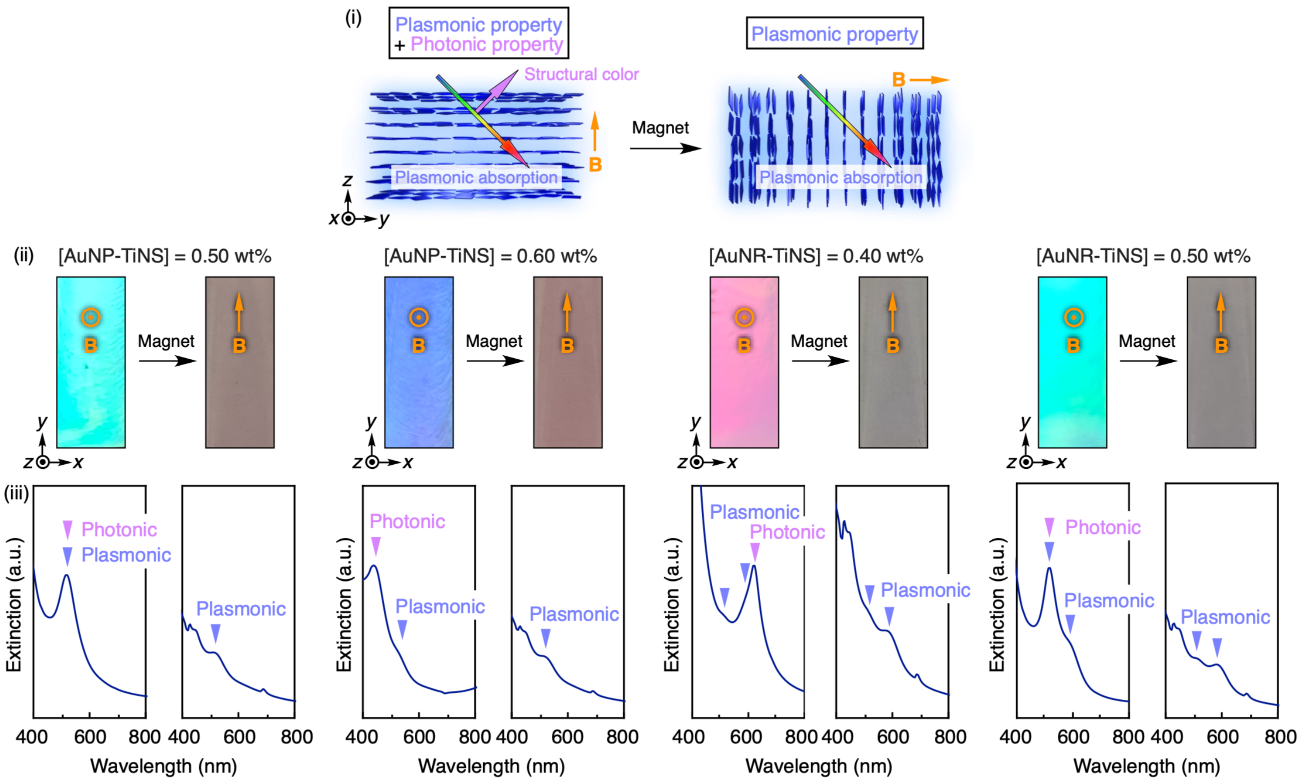

Supplementary Fig. 19 | Magneto-responsive structural colors of the multi-functional photonic crystals.

(i) Schematic illustrations, (ii) optical images, and (iii) extinction spectra of the photonic crystals of AuNP-TiNSs (0.50 and 0.60 wt%) and AuNR-TiNSs (0.40 and 0.50 wt%) by changing the direction of the applied magnetic field from the *z*-axis (left) to the *y*-axis (right).

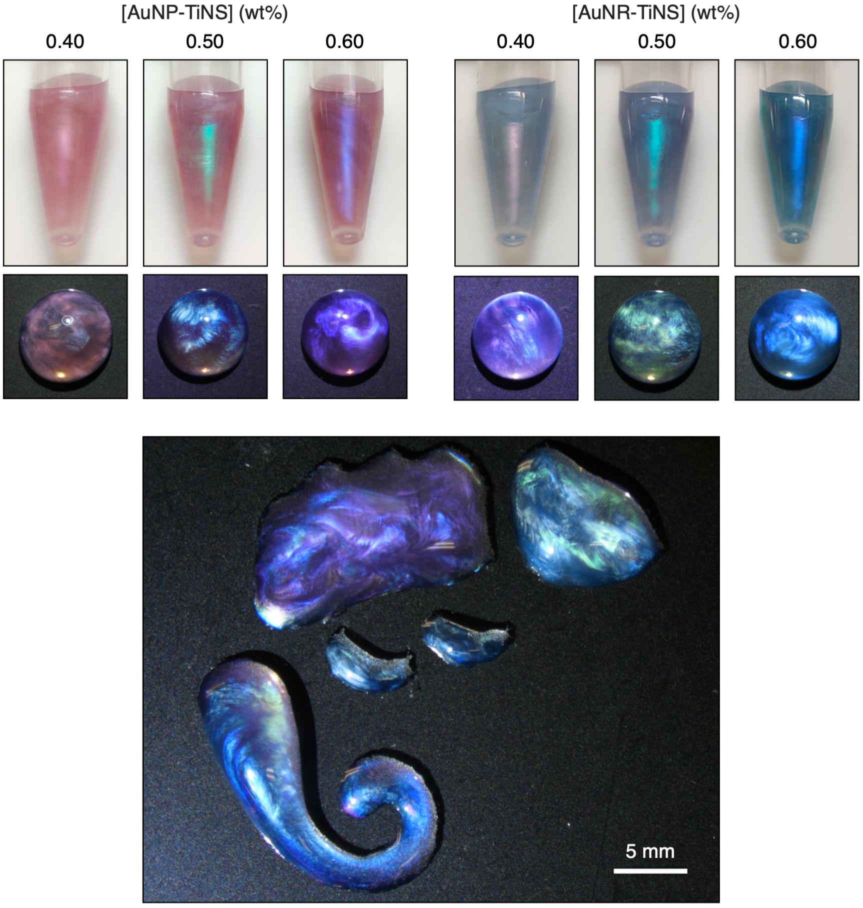

Supplementary Fig. 20 | Photonic inks from the multi-functional photonic crystals.

Optical images of the photonic crystals of AuNP-TiNSs and AuNR-TiNSs (0.40, 0.50, and 0.60 wt%) and demonstration as photonic inks with reflection- and absorption-based coloration.

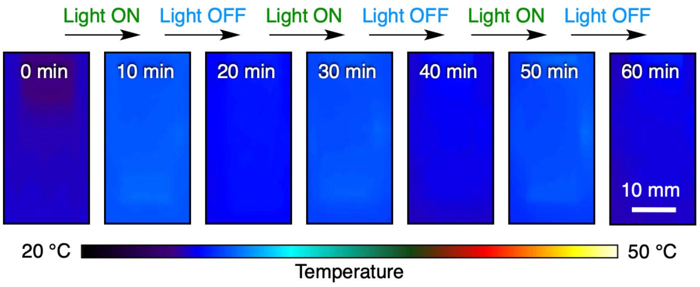

Supplementary Fig. 21 | Thermal images of the photonic crystal of TiNSs upon light irradiation. Thermal images of the magnetically treated photonic crystal of pristine TiNSs (0.60 wt%) before and after 10 min of green light irradiation.
