# Supplementary material for: Multi-functional photonic crystals of modular nanosheets
Source: Nat Commun. 2026 May 26;17:4517. doi: 10.1038/s41467-026-70456-6 (PMC13212688; doi:10.1038/s41467-026-70456-6)
Supplement: Supplementary file 2 — Description of Additional Supplementary Information [file 41467_2026_70456_MOESM2_ESM.pdf]

Description of Additional Supplementary Files

File Name: Supplementary Movie 1
Description: Fluorescence microscopy movie of fluorescent nanosheets.
Fluorescence microscopy movie (10× speed) of an aqueous dispersion of FSNP-TiNSs (0.0050 wt%).

File Name: Supplementary Movie 2
Description: CLSM movie of the magnetically treated photonic crystal of FSNP-TiNSs.
CLSM movie (500× speed) of the magnetically treated photonic crystal of FSNP-TiNSs (0.40 wt%) in a dispersion state using a 550-nm laser.

File Name: Supplementary Movie 3
Description: CLSM movie of the photonic crystal of FSNP-TiNSs within giant vesicles.
CLSM overlay movie (40× speed) of the photonic crystal of FSNP-TiNSs (0.40 wt%) within giant vesicles using a 460-nm laser for the vesicles and a 550-nm laser for the nanosheets.
